# Supplementary material for: Perceived Supervisor Support for Health Affects Presenteeism: A Cross-Sectional Study
Source: Int J Environ Res Public Health. 2022 Apr 4;19(7):4340. doi: 10.3390/ijerph19074340 (PMC8998755; doi:10.3390/ijerph19074340)
Supplement: Supplementary file 1 [file ijerph-19-04340-s001.zip › ijerph-1630019-supplementary.pdf]

International Journal of
Environmental Research and Public Health

Table S1. Relationship between perceived supervisor support for health as a continuous variable and the top 10% by presenteeism scores (a score of 64 or higher).

	Model 1			Model 2			Model 3			Model 4		
	OR	95% CI	*p*-Value	aOR	95% CI	*p*-Value	aOR	95% CI	*p*-Value	aOR	95% CI	*p*-Value
PSSH (continuous)	1.53	1.44–1.63	< 0.001	1.54	1.45–1.64	< 0.001	1.20	1.12–1.29	< 0.001	1.07	1.00–1.15	0.056
K6 score (continuous)							1.14	1.13–1.15	< 0.001	1.12	1.11–1.14	< 0.001
WE score (continuous)										0.97	0.96–0.98	< 0.001

Model 1: crude model. Model 2: adjusted for age, gender, and occupation. Model 3: Model 2, additionally adjusted for K6 score. Model 4: Model 3, additionally adjusted for work engagement score. All analyses used multilevel logistic regression nested by company. PSSH: perceived supervisor support for health; WE: work engagement; aOR: adjusted odds ratio; CI: confidence interval.

Table S2. Relationship between perceived supervisor support for health as a categorical variable and the top 10% by presenteeism scores (a score of 64 or higher).

	Model 1			Model 2			Model 3			Model 4		
	OR	95% CI	*p*-Value	aOR	95% CI	*p*-Value	aOR	95% CI	*p*-Value	aOR	95% CI	*p*-Value
PSSH (categorical)												
Very high	Ref			Ref			Ref			Ref		
High	1.27	1.10–1.47	0.001	1.29	1.12–1.49	< 0.001	1.11	0.95–1.28	0.181	0.95	0.82–1.11	0.538
Low	2.13	1.81–2.51	< 0.001	2.17	1.84–2.55	< 0.001	1.45	1.22–1.72	< 0.001	1.13	0.94–1.35	0.194
Very low	3.39	2.73–4.20	< 0.001	3.46	2.78–4.30	< 0.001	1.61	1.27–2.04	< 0.001	1.14	0.89–1.46	0.302
K6 score (continuous)							1.14	1.13–1.15	< 0.001	1.12	1.11–1.14	< 0.001
WE score (continuous)										0.97	0.96–0.97	< 0.001

Model 1: crude model. Model 2: adjusted for age, gender, and occupation. Model 3: Model 2, additionally adjusted for K6 score. Model 4: Model 3, additionally adjusted for work engagement score. All analyses used multilevel logistic regression nested by company. PSSH: perceived supervisor support for health; WE: work engagement; aOR: adjusted odds ratio; CI: confidence interval.

Table S3. Relationship between perceived supervisor support for health as a continuous variable and the top 30% by presenteeism scores (a score of 30 or higher).

	Model 1			Model 2			Model 3			Model 4		
	OR	95% CI	*p*-Value	aOR	95% CI	*p*-Value	aOR	95% CI	*p*-Value	aOR	95% CI	*p*-Value
PSSH (continuous)	1.54	1.47–1.62	< 0.001	1.56	1.48–1.63	< 0.001	1.27	1.21–1.34	< 0.001	1.18	1.12–1.24	< 0.001
K6 score (continuous)							1.14	1.13–1.15	< 0.001	1.13	1.12–1.14	< 0.001
WE score (continuous)										0.98	0.97–0.98	< 0.001

Model 1: crude model. Model 2: adjusted for age, gender, and occupation. Model 3: Model 2, additionally adjusted for K6 score. Model 4: Model 3, additionally adjusted for work engagement score. All analyses used multilevel logistic regression nested by company. PSSH: perceived supervisor support for health; WE: work engagement; aOR: adjusted odds ratio; CI: confidence interval.

Table S4. Relationship between perceived supervisor support for health as a categorical variable and the top 30% by presenteeism scores (a score of 30 or higher).

	**Model 1**			**Model 2**			**Model 3**			**Model 4**		
	**OR**	**95% CI**	***p*-Value**	**aOR**	**95% CI**	***p*-Value**	**aOR**	**95% CI**	***p*-Value**	**aOR**	**95% CI**	***p*-Value**
PSSH (categorical)												
Very high	Ref			Ref			Ref			Ref		
High	1.42	1.29–1.56	< 0.001	1.44	1.30–1.58	< 0.001	1.25	1.13–1.38	< 0.001	1.13	1.02–1.25	0.025
Low	2.35	2.09–2.64	< 0.001	2.40	2.13–2.70	< 0.001	1.70	1.50–1.92	< 0.001	1.43	1.26–1.63	< 0.001
Very low	3.49	2.93–4.15	< 0.001	3.60	3.02–4.29	< 0.001	1.88	1.56–2.28	< 0.001	1.50	1.23–1.82	< 0.001
K6 score (continuous)							1.14	1.13–1.15	< 0.001	1.13	1.12–1.14	< 0.001
WE score (continuous)										0.98	0.97–0.98	< 0.001

Model 1: crude model. Model 2: adjusted for age, gender, and occupation. Model 3: Model 2, additionally adjusted for K6 score. Model 4: Model 3, additionally adjusted for work engagement score. All analyses used multilevel logistic regression nested by company. PSSH: perceived supervisor support for health; WE: work engagement; aOR: adjusted odds ratio; CI: confidence interval.
